# Development and evaluation of a Register-Based Organ Damage Index in systemic lupus erythematosus: a nationwide, population-based study from Sweden

**DOI:** 10.1136/lupus-2024-001403

**Published:** 2025-02-26

**Authors:** Alvaro Gomez, Ioannis Parodis, Muna Saleh, Julia F Simard, Christopher Sjöwall, Elizabeth V Arkema

**Affiliations:** 1 Division of Rheumatology, Department of Medicine Solna, Karolinska Institutet, Stockholm, Sweden; 2 Department of Rheumatology, Faculty of Medicine and Health, Örebro universitet, Örebro, Sweden; 3 Department of Biomedical and Clinical Sciences, Division of Inflammation and Infection/Rheumatology, Linköpings universitet, Linköping, Sweden; 4 Department of Epidemiology and Population Health; Division of Immunology and Rheumatology, Department of Medicine, Stanford University School of Medicine, Stanford, California, USA; 5 Clinical Epidemiology Division, Department of Medicine Solna, Karolinska Institutet, Stockholm, Sweden

**Keywords:** Systemic Lupus Erythematosus, Epidemiology, Mortality, Outcome Assessment, Health Care, Sensitivity and Specificity

## Abstract

**Objective:**

To develop a Register-Based Organ Damage Index (RBODI) in SLE, and evaluate its accuracy in estimating Systemic Lupus International Collaborating Clinics/American College of Rheumatology (SLICC/ACR) Damage Index (SDI) scores. Additionally, to describe organ damage accrual and associations with mortality in a Swedish population-based nationwide cohort.

**Methods:**

SDI items were translated into diagnosis, treatment and procedural codes retrieved from Swedish health registers. RBODI was calculated using the same rules as the SDI and its accuracy was evaluated using SDI data from the *Clinical Lupus Register in North-Eastern Gothia* cohort as the gold standard. Among newly diagnosed patients with SLE from Sweden (2005–2021), we estimated 5-year risks of organ damage, and adjusted HRs of first RBODI-based organ damage accrual associated with patient characteristics. Lastly, we estimated the association between RBODI-based organ damage within 5 years of diagnosis and mortality.

**Results:**

The evaluation cohort included 271 prevalent cases (65.3% developed organ damage). RBODI had a positive predictive value of 90%, sensitivity 80% and specificity 83%. Among 4441 newly diagnosed patients with SLE, 40% developed organ damage within 5 years. Males had a 30% higher risk of developing damage compared with females (HR 1.3) and older individuals (>45 years old compared with younger) had more than threefold higher risk (HR 3.3). Early development of organ damage was associated with a 2.1-fold higher risk of mortality.

**Conclusion:**

Our novel RBODI accurately estimates SDI scores and describes long-term trends in damage accrual in the largest cohort of incident SLE to date. The strong association between early damage accrual and mortality highlights the need for efficient prevention strategies.

**WHAT IS ALREADY KNOWN ON THIS TOPIC**

- Prevention of organ damage is one of the key therapeutic goals in SLE. The gold standard to evaluate organ damage is the Systemic Lupus International Collaborating Clinics/American College of Rheumatology (SLICC/ACR) Damage Index (SDI), which is an extensively validated instrument. SDI has mainly been used in selected populations from specialised SLE centres and in randomised controlled trials without long-term follow-up, hindering much-needed real-world investigations of interventions on organ damage accrual.

**WHAT THIS STUDY ADDS**

- We developed a Register-Based Organ Damage Index (RBODI) by translating codes from healthcare interactions into SDI items, and demonstrated that RBODI accurately detects organ damage as scored with SDI.
- In the largest cohort of incident SLE to date, we observed that 40% of patients develop organ damage within the first 5 years since diagnosis. Individuals who accrued damage within the first 5 years of diagnosis have a substantially increased risk of mortality.
- Over the last two decades, there have been no changes in the risk of organ damage for newly diagnosed patients with SLE by calendar period.

**HOW THIS STUDY MIGHT AFFECT RESEARCH, PRACTICE OR POLICY**

- RBODI offers an efficient way to generate real-world evidence on the effect of interventions in the prevention of organ damage.
- Our findings highlight the unmet need for effective strategies to prevent organ damage in the SLE population.

## Introduction

 One of the key therapeutic goals in SLE is the prevention of organ damage.^1 2^ Organ damage denotes decline or loss of function as a consequence of the inflammatory activity of the disease, treatment toxicity, comorbidities, ageing and/or incidental events.^3^ It is irreversible and tends to increase over time. Importantly, organ damage is a measure of morbidity that is tightly linked with the risk of mortality.^4^

Accrual of damage is assessed using the Systemic Lupus International Collaborating Clinics/American College of Rheumatology (SLICC/ACR) Damage Index (SDI).^5^ Designed in 1996, and extensively validated,^6 7^ the SDI is a weighted scale that comprises 41 items grouped into 12 domains. To be scored, damage in an item must have developed after the SLE diagnosis, be present for at least 6 months for most items and confirmed by clinical evaluation. The SDI score is positively associated with higher risk of mortality,^4 8 9^ further development of organ damage^4 10 11^ and poor health-related quality of life.^4 12 13^

The SDI is an important measurement of disease burden, but it has mainly been used in selected populations from specialised SLE centres and in randomised controlled trials (RCTs) without long-term follow-up. As organ damage develops slowly, regular follow-up over time is required to detect the accrual of organ damage. Therefore, access to long-term longitudinal organ damage data on large populations of unselected patients with SLE would enable much-needed real-world investigations of interventions on organ damage accrual.

We developed a novel Register-Based Organ Damage Index (RBODI) using administrative codes from healthcare interactions and aimed to evaluate its accuracy in estimating SDI scores collected from a well-characterised clinical cohort. Furthermore, we described organ damage accrual and its association with mortality using RBODI in newly diagnosed patients with SLE in a population-based nationwide cohort from Sweden.

## Methods

### Study design

We conducted a validation study to evaluate the accuracy of RBODI to estimate SDI scores overall and within different organ domains. We also performed a population-based cohort study to describe the occurrence of organ damage using RBODI in patients with incident SLE in Sweden.

### Data sources

We linked several national population-based Swedish registers to define the study populations and to construct the RBODI. The National Patient Register covers inpatient care since 1969 (national coverage from 1987) and specialist outpatient care since 2001. Primary and secondary diagnoses are coded according to the Swedish version of the International Classification of Diseases (ICD)-9 (1973–1996) and ICD-10 (since 1997); medical procedures are coded according to Klassifikation av vårdåtgärder (KVÅ) codes; diagnoses and procedures are further coded according to the Diagnosis Related Group (DRG) codes. The Prescribed Drug Register contains information on dispensations of prescribed drugs from pharmacies since July 2005, coded according to the Anatomical Therapeutic Chemical (ATC) codes. The Total Population Register contains information on date of birth and migration, and the Cause of Death Register records date of death.

The *Clinical Lupus Register in North-Eastern Gothia* (KLURING) cohort at Linköping University Hospital is a regional register of adults with SLE diagnosed according to the 1982 ACR^14^ and/or 2012 SLICC^15^ classification criteria, as the cohort was established before the 2019 EULAR/ACR classification criteria had been proposed. This register has enrolled prevalent and incident cases since 2008, covering more than 97% of SLE cases from the Östergötland County, as previously described.^16 17^

### Construction of RBODI

We used information on clinical outpatient or inpatient diagnoses and procedures from the National Patient Register, as well as dispensed medications from the Prescribed Drug Register to estimate organ damage accrual, by leveraging register-based codes from healthcare interactions into SDI items. These different systems allowed us to use medical diagnoses (ICD-10), medical and surgical procedures (retrieved from KVÅ and DRG codes, respectively) and dispensed medications (ATC) to identify SDI items. Considering that these codes are provided by practitioners from all specialties, we developed the translation in collaboration with physicians from the cardiology, gastroenterology, internal medicine, neurology, ophthalmology, pulmonology, psychiatry and rheumatology departments, including several physicians experienced in the care of patients with SLE . Several diagnoses included as SDI items have been previously validated in Sweden; in these instances, the validated definitions were adopted (references appended in online supplemental table S1). For diagnoses lacking prior validation, expert opinion guided the formulation of the definitions. We followed the principles in the SDI scoring system, using the same weighting system, and scored damage occurring since SLE diagnosis by requiring each RBODI item to occur since the date of the first SLE-coded visit for every individual. We incorporated the 6-month criterion by either using codes indicative of chronicity (eg, N18.5: chronic kidney disease, stage 5), or requiring the presence of two or more codes with a minimum interval of 6 months between registrations (eg, L97: ulcer of lower limb, not elsewhere classified). As repeated administrative codes for the same diagnosis do not necessarily reflect repeated episodes for such diagnosis, we refrained from scoring repeated SDI items, except for myocardial infarction (ICD-10 code I22: subsequent myocardial infarction).

### Study populations

#### Evaluation cohort

The KLURING cohort was linked to nationwide Swedish registers and restricted to individuals with an SLE diagnosis up to 2021, living in Östergötland County in 2021. None of these patients had missing SDI data. Online supplemental figure S1 shows the flow diagram for the selection of participants.

In patients from this well-characterised cohort used for evaluation, organ damage was assessed annually by the same experienced rheumatologist (CS) using the SDI. The SDI was calculated with information retrieved during the medical appointments and complemented with manual chart review, when appropriate.

#### Nationwide incident SLE cohort

We used the National Patient Register to identify adults receiving at least two ICD-10-coded visits with a diagnosis of SLE (M32, excluding M32.0, drug-induced SLE) within 1 year, including at least one with a specialist in rheumatology, dermatology, nephrology, internal medicine or paediatrics. This method has been shown to have high accuracy.^18^ To restrict to incident SLE cases, we required the first SLE diagnosis to occur on 1 July 2005 or later (providing a 4-year period with available outpatient data and extensive inpatient data to exclude prevalent SLE cases) and that individuals had lived in Sweden for at least 2 years before the first SLE diagnosis (aiming to exclude prevalent cases who have migrated to Sweden).

### Ethics

All study participants included in the KLURING cohort provided informed consent, whereas consent from individuals identified exclusively through the registers is not required in Sweden. Ethical permission for the present investigation was granted by the Swedish Ethical Review Authority (reference: 2021-01148) and the Regional Ethics Board in Linköping (reference: M75-08).

### Statistical analysis

We described the characteristics of both study populations using frequencies and median (IQR). Given that the evaluation cohort had complete SDI data for all patients with SLE living in the Östergötland county in 2021, we estimated sensitivity (Se), specificity (Sp), positive predictive value (PPV) and negative predictive value (NPV) of RBODI to detect the presence of organ damage (SDI=0 vs SDI>0), using SDI data from KLURING as the gold standard. The estimation of 95% CI for the validation parameters assumed a normal approximation to the binomial distribution. Additionally, we estimated the same parameters to measure the accuracy of RBODI across different organ domains, as well as its accuracy in detecting damage related to and independent of glucocorticoids, as defined by Gladman *et al*.^19^ As a complementary measure, we evaluated the agreement between RBODI and SDI scores by estimating a kappa coefficient with quadratic weighting along with 95% CI, given that both RBODI and SDI are ordinal variables. The study design for the validation of RBODI is depicted in online supplemental figure S2.

In the nationwide incident SLE cohort, we estimated the 5-year cumulative incidence of any organ damage overall (RBODI>0), and by patient characteristics (age, sex and year of diagnosis), using Kaplan-Meier estimation. To account for differences in follow-up time, we used Cox models to estimate the age-adjusted and sex-adjusted HR of first organ damage development (RBODI>0) within 5 years from the SLE diagnosis associated with patient characteristics. For these analyses, patients were followed up from the date of first SLE-coded visit until the date of development of first organ damage, 5 years of follow-up, emigration, death or end of follow-up on 31 December 2021, whichever came first. Online supplemental figure S3 depicts the study design for the occurrence of organ damage.

Lastly, we aimed to compare with a previous report from Sweden^9^ that explored the association between SDI score at 5 years from diagnosis and long-term mortality, by estimating age-adjusted and sex-adjusted HR of mortality associated with (1) any organ damage and (2) continuous RBODI score within the first 5 years of diagnosis, in a subcohort of incident SLE in which individuals were required to be living in Sweden for at least 5 years from the SLE diagnosis (n=2880). Online supplemental figure S4 depicts the study design for the analysis of mortality.

We used the SAS statistical software V.9.4 (SAS Institute, Cary, North Carolina, USA) for data management, and R V.4.3.1 (R Foundation for Statistical Computing, Vienna, Austria) for data management and statistical analysis.

## Results

### Development of the RBODI

The detailed translation of SDI items into the RBODI is presented in online supplemental table S1. The majority of the index relies on ICD-10 codes. In the nationwide incident SLE population, we could observe at least one occurrence of all diagnoses included in the SDI, except for shrinking lung, mesenteric insufficiency, pancreatic insufficiency, and extensive scarring of panniculum other than scalp and pulp space.

### Evaluation cohort description

Out of 332 patients with SLE enrolled in KLURING since 2008, the evaluation cohort included 271 individuals living in Östergötland in 2021. About 87% were female, with a median age at diagnosis of 39 (IQR: 28.0–53.1) years and a median disease duration of 16.1 (IQR: 9.3–20.1) years. Most individuals had ever presented with musculoskeletal, mucocutaneous and haematological manifestations, and nearly one-third had renal disease (table 1).

**Table 1:** Demographic and clinical characteristics of SLE cases from Östergötland, Sweden 2021 (evaluation cohort)

	Evaluation cohort (n=271)
Demographics	
Age at diagnosis (years), median (IQR)	39.0 (28.0–53.1)
Female sex, n (%)	235 (86.7)
Disease duration (years), median (IQR)	16.1 (9.3–20.1)
Level of education (years), n (%)	
0–9	63 (23.2)
10–12	106 (39.1)
13 or more	71 (26.2)
Missing	31 (11.4)
Clinical manifestations (1982 ACR criteria definitions)	
Number of ACR criteria, median (IQR)	5.0 (4.0–6.0)
Malar rash, n (%)	101 (37.3)
Discoid lupus, n (%)	37 (13.7)
Photosensitivity, n (%)	138 (50.9)
Oral ulcers, n (%)	36 (13.3)
Arthritis, n (%)	209 (77.1)
Serositis, n (%)	92 (33.9)
Renal disorder, n (%)	80 (29.5)
Neurological disorder, n (%)	18 (6.6)
Haematological disorder, n (%)	168 (62.0)
Immunological disorder, n (%)	156 (57.6)
Antinuclear antibody, n (%)	268 (98.9)
Medication dispensed from pharmacy during 2021	
Antimalarial agents, n (%)	187 (69.0)
Glucocorticoids, n (%)	142 (52.4)
Azathioprine, n (%)	14 (5.2)
Methotrexate, n (%)	26 (9.6)
Mycophenolic acid, n (%)	41 (15.1)
Belimumab subcutaneous, n (%)	10 (3.7)
Cyclosporine, n (%)	2 (0.7)

ACR, American College of Rheumatology

### Accuracy of RBODI

**Figure 1:**
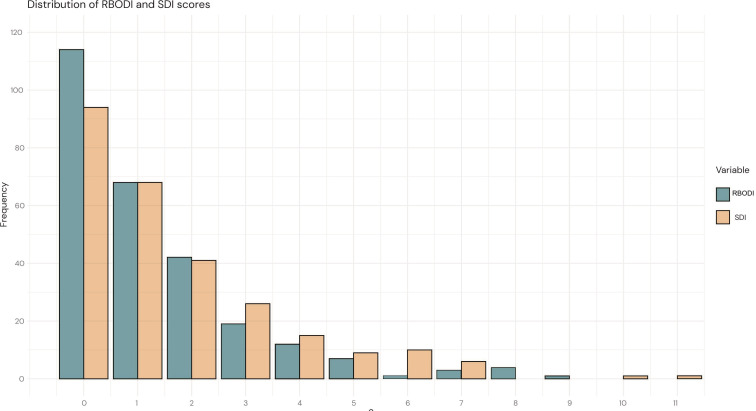
Distribution of SDI and RBODI scores in SLE cases from Östergötland, Sweden 2021 (validation cohort; n=271). RBODI, register-based damage organ index; SDI, SLICC/ACR Damage Index.

Organ damage in 2021, assessed with SDI or estimated with RBODI, along with the validation parameters for RBODI, are summarised in table 2. The proportion of patients who had any damage was 177/271 (65.3%) according to the SDI, and 157/271 (57.9%) according to RBODI. The score distributions for both SDI and RBODI were positively skewed and closely resembled each other (figure 1), with approximately 90% of patients scoring 4 or less on SDI and 94% on RBODI.

**Table 2:** Accuracy measurements of RBODI to detect the presence of organ damage according to SDI in SLE cases from Östergötland, Sweden 2021 (n=271)

Item	RBODI,n (%)	SDI,n (%)	Se(95% CI)	Sp(95% CI)	PPV(95% CI)	NPV(95% CI)
Any organ damage	157 (57.9)	177 (65.3)	0.80(0.73, 0.85)	0.83(0.74, 0.90)	0.90(0.84, 0.94)	0.68(0.59, 0.77)
Organ damage >1	89 (32.8)	109 (40.2)	0.71(0.61, 0.79)	0.93(0.87, 0.96)	0.87(0.78, 0.93)	0.82(0.76, 0.88)
Any organ damage across organ domains						
Ocular	61 (22.5)	49 (18.1)	0.90(0.78, 0.97)	0.92(0.88, 0.95)	0.72(0.59, 0.83)	0.98(0.95, 0.99)
Neuropsychiatric	35 (12.9)	63 (23.4)	0.48(0.35, 0.61)	0.98(0.94, 0.99)	0.86(0.70, 0.95)	0.86(0.81, 0.90)
Renal	10 (3.7)	16 (6.0)	0.56(0.30, 0.80)	1.00(0.98, 1.00)	0.90(0.55, 1.00)	0.97(0.95, 0.99)
Pulmonary	17 (6.3)	24 (8.9)	0.54(0.33, 0.74)	0.98(0.96, 1.00)	0.76(0.50, 0.93)	0.96(0.92, 0.98)
Cardiovascular	38 (14.0)	47 (17.3)	0.72(0.57, 0.84)	0.98(0.95, 1.00)	0.89(0.75, 0.97)	0.94(0.91, 0.97)
Peripheral vascular	27 (10.0)	26 (9.6)	0.69(0.48, 0.86)	0.96(0.93, 0.98)	0.67(0.46, 0.83)	0.97(0.94, 0.99)
Gastrointestinal	23 (8.5)	22 (8.1)	0.77(0.55, 0.92)	0.98(0.95, 0.99)	0.74(0.52, 0.90)	0.98(0.95, 0.99)
Musculoskeletal	34 (12.5)	55 (20.3)	0.35(0.22, 0.49)	0.99(0.96, 1.00)	0.86(0.65, 0.97)	0.86(0.81, 0.90)
Mucocutaneous	3 (1.1)	15 (5.5)	0.00(0.00, 0.22)	0.99(0.97, 1.00)	0.00(0.00, 0.71)	0.94(0.91, 0.97)
Premature gonadal failure	2 (0.7)	4 (1.5)	0.00(0.00, 0.60)	0.99(0.97, 1.00)	0.00(0.00, 0.84)	0.99(0.96, 1.00)
Diabetes	15 (5.5)	16 (5.9)	0.88(0.62, 0.98)	1.00(0.98, 1.00)	0.93(0.68, 1.00)	0.99(0.97, 1.00)
Malignancy	30 (11.8)	34 (12.5)	0.93(0.78, 0.99)	0.98(0.95, 0.99)	0.82(0.65, 0.93)	0.99(0.97, 1.00)

NPV, negative predictive value; PPV, positive predictive value; RBODI, Register-Based Organ Damage Index; SDI, SLICC/ACR Damage Index; Se, sensitivity; Sp, specificity

Overall, the RBODI showed high accuracy for detecting the presence of organ damage (SDI=0 vs SDI>0), with Se: 0.80 (95% CI 0.73 to 0.85), Sp: 0.83 (95% CI 0.74 to 0.90), PPV: 0.90 (95% CI 0.84 to 0.94) and NPV: 0.68 (95% CI 0.59 to 0.77). Among the 36/271 individuals who had acquired organ damage according to SDI but not RBODI (false negatives), the organ domains most commonly involved were neuropsychiatric (n=11; mostly driven by six cases of cognitive dysfunction), mucocutaneous (n=7) and musculoskeletal (n=7).

When looking across organ domains, Sp and NPV were consistently very high, and there were substantial differences in Se and PPV. The largest Se values were observed in the ocular, malignancy and diabetes domains (all >0.85), while the largest PPV values were observed in the diabetes, renal, cardiovascular, neuropsychiatric and musculoskeletal domains (all >0.85). Damage in the mucocutaneous and premature gonadal failure domains could not be classified accurately using RBODI.

The validation parameters for RBODI to detect damage related to glucocorticoids were similar to those for overall damage, with a slight increase in Sp (0.88; 95% CI 0.82 to 0.93) and NPV (0.80; 95% CI 0.73 to 0.86), while Se was lower for detecting glucocorticoid-independent damage (0.61; 95% CI 0.51 to 0.71; online supplemental table S2).

We observed a high level of agreement between RBODI and SDI scores with a weighted kappa coefficient of 0.80 (95% CI 0.68 to 0.92).

### Development of organ damage in the nationwide cohort of newly diagnosed patients with SLE

After developing and validating the RBODI using SDI from the evaluation cohort as the gold standard, we analysed the occurrence of RBODI-measured organ damage in a nationwide incident SLE cohort comprising 4441 individuals. In this cohort, 82.4% were female, and the median age at the time of diagnosis was 47.1 (IQR: 31.3–63.9) years. The median interval between the first SLE-coded visit and reaching criteria for inclusion was 1.6 (IQR: 0.6–3.8) months. During the year prior to the first SLE-coded visit, glucocorticoids (41.5%) and antimalarial agents (19.1%) were the drug classes most commonly dispensed (table 3).

**Table 3:** Demographic and clinical characteristics of newly diagnosed SLE cases from Sweden, 2005–2021 (incident nationwide cohort)

	Incident cohort (N=4441)
Demographics	
Age at diagnosis (years), median (IQR)	47.1 (31.3–63.9)
Female sex, n (%)	3661 (82.4)
Disease duration (months), median (IQR)	1.6 (0.6–3.8)
Level of education (years), n (%)	
0–9	996 (22.4)
10–12	1693 (38.1)
13 or more	1307 (29.4)
Missing	445 (10.0)
Region of residence, n (%)	
North	322 (7.3)
South	692 (15.6)
Southeast	466 (10.5)
Stockholm	924 (20.8)
Uppsala-Örebro	911 (20.5)
West	756 (17.0)
Missing	370 (8.3)
Clinical manifestations	
Number of hospitalisations the prior year, n (%)	
0	2814 (63.4)
1–3	1404 (31.6)
4 or more	223 (5.0)
Medication dispensed from the pharmacy during the prior year	
Antimalarial agents, n (%)	848 (19.1)
Glucocorticoids, n (%)	1844 (41.5)
Azathioprine, n (%)	165 (3.7)
Methotrexate, n (%)	275 (6.2)
Mycophenolic acid, n (%)	64 (1.4)
Cyclosporine, n (%)	25 (0.6)

During the first 5 years after SLE diagnosis, 39.7% of patients with SLE developed organ damage in at least one item. The 5-year cumulative incidence of organ damage ranged from 0.2% (95% CI 0.1% to 0.3%) to 14.0% (95% CI 12.9% to 15.2%) across the different organ domains. The largest cumulative incidences were observed for the ocular, neuropsychiatric and cardiovascular domains (online supplemental table S3 and figure S5).

**Figure 2:**
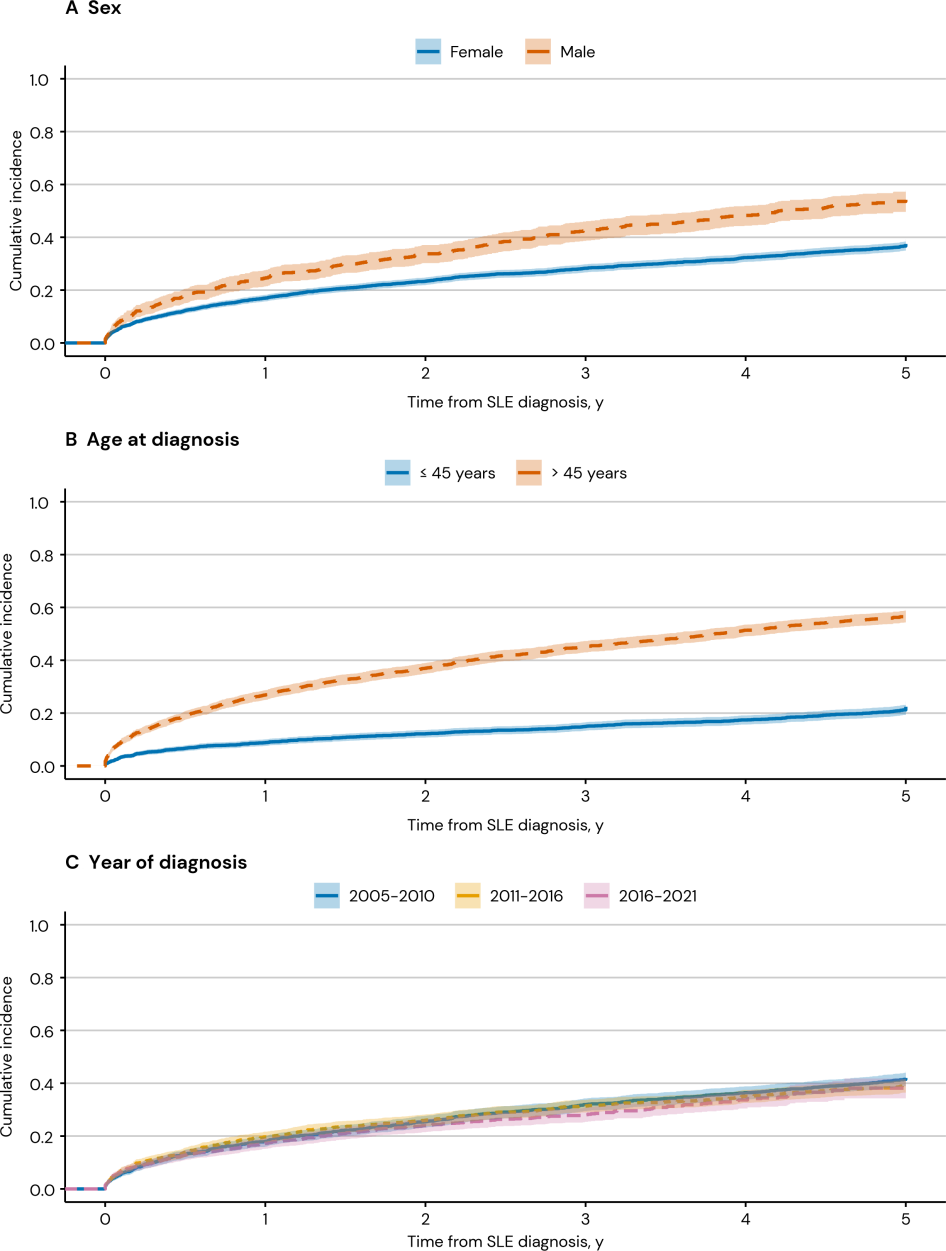
Cumulative incidence of organ damage accrual in newly diagnosed patients with SLE in Sweden (n=4441).

The 5-year cumulative incidence of organ damage was higher among men compared with women, and among older compared with younger individuals (figure 2). The age-adjusted HR of any organ damage accrual for men versus women was 1.30 (95% CI 1.16 to 1.47), while the sex-adjusted HR for older versus younger individuals (>45 years vs ≤45 years) was 3.30 (95% CI 2.94 to 3.69). Calendar year of diagnosis was not associated with organ damage accrual, indicating that the risk of organ damage remained stable over time (HR 0.99; 95% CI 0.98 to 1.00).

### Associations between organ damage accrual and mortality risk in the nationwide cohort of newly diagnosed patients with SLE

**Figure 3:**
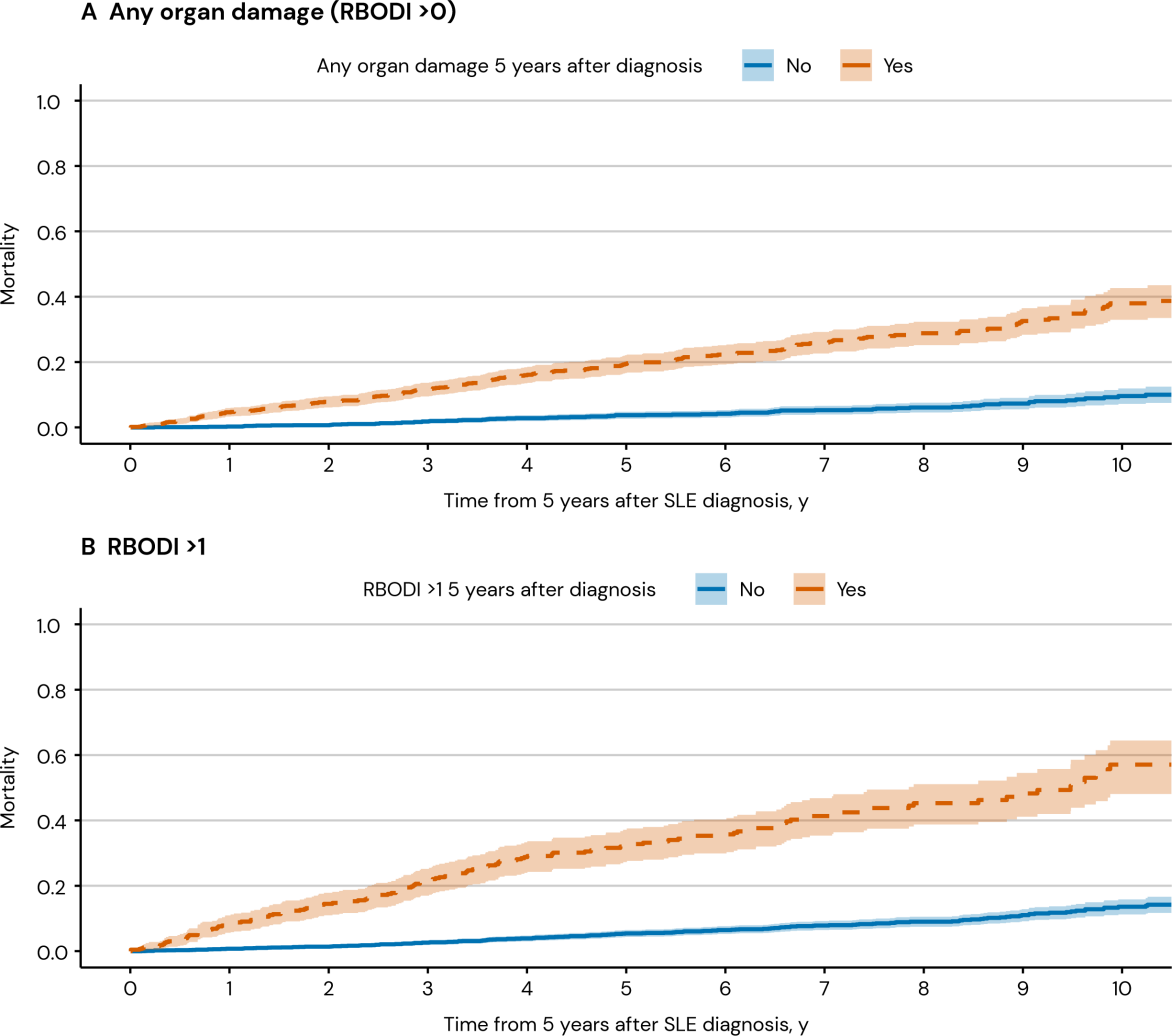
Mortality from 5 years after diagnosis in newly diagnosed patients with SLE in Sweden (n=2880). RBODI, Register-Based Damage Organ Index.

We identified 2880 newly diagnosed patients with SLE living in Sweden for at least 5 years after their SLE diagnosis. Among them, 335 deaths were recorded during a median follow-up time of 10.1 (IQR: 7.6–13.3) years. Compared with individuals who did not develop organ damage during the first 5 years of diagnosis, those with an RBODI score >0 during the same study period had a 2.1-fold higher age-adjusted and sex-adjusted hazard of mortality (HR 2.14; 95% CI 1.67 to 2.73; figure 3). The adjusted HR comparing patients with RBODI scores >1 versus ≤1 was 2.35 (95% CI 1.88 to 2.94). The RBODI score at 5 years from diagnosis as a continuous measure was associated with an increased age-adjusted and sex-adjusted hazard of mortality (HR 1.40; 95% CI 1.32 to 1.49).

## Discussion

Despite an increasing understanding of the comorbidity burden in patients with SLE,^20 21^ this is, to our knowledge, the first study to develop a register-based measurement of organ damage in SLE and assess its accuracy. RBODI displayed high accuracy to detect organ damage, with a Se of 80%, Sp of 83% and a PPV of 90%. When applied in a nationwide cohort of newly diagnosed patients with SLE from Sweden, 40% developed organ damage within 5 years from the SLE diagnosis, consistent with previous reports using SDI data from Sweden,^9 16^ the UK^22^ and the global SLICC cohort.^4 23^ Men and older individuals had a higher risk of developing organ damage than their counterparts, while no change in the risk of organ damage across calendar periods was observed. Lastly, in line with earlier observations, there was a strong association between early development of RBODI-based organ damage and long-term mortality.

Previous studies have found a high agreement between SDI scores retrieved from manual chart review and prospectively collected SDI scores.^24 25^ Consistent with our study, the highest agreement between SDI scores and manual chart review^24 25^ was for the ocular, malignancy and diabetes domains, while the mucocutaneous and premature gonadal failure were the domains less accurately captured. Similar trends were reported when organ damage was evaluated using patient-reported data.^26^

Previous studies have reported that 35%–54% of patients develop organ damage measured with SDI within the first 5 years from diagnosis.^4 9 16^ Estimates differ across studies, which may be due to the heterogeneity in disease presentation and healthcare practices across different cohorts. In our study, we observed a 5-year cumulative incidence of 40% in patients with SLE from the entire country of Sweden, closely resembling the figure reported from Östergötland county, Sweden (42%).^16^ Given the chronic course of SLE, coupled with the adverse outcomes associated with the occurrence of damage, we show that organ damage accrual remains frequent. Notably, despite advancements in medical care and updates in clinical guidelines,^1 2^ we did not observe a reduction in the risk of damage across calendar periods. These findings underscore the ongoing need for multimodal strategies to prevent organ damage in SLE. Determining whether new promising treatments will prevent organ damage remains a key focus in the research agenda; additionally, the relationship between current therapies, such as glucocorticoids, and organ damage warrants further investigation.

Beyond accurately estimating SDI scores, the reliability of RBODI is demonstrated by the expected associations between male sex and older age and RBODI-assessed organ damage. As consistently shown with SDI data,^27^ this is expected as both instruments measure morbidity influenced not only by disease activity, but also drug toxicity, comorbidities, ageing and incidental events.^3^ These trends are likely observable in other diseases and in the general population.^28^

Organ damage estimated using the SDI has consistently been associated with increased mortality in SLE, a relationship that has been pivotal in confirming the validity of SDI as a measure of organ damage.^29^ Most studies that have evaluated SDI as a continuous predictor have found an increased risk of mortality, with HRs ranging from 1.2 to 1.5.^30^ Studies evaluating SDI as a binary predictor had different inclusion criteria and used different cut-offs and statistical approaches. Previous research from Sweden comparing SDI scores >1 versus ≤1 reported a relative risk of 3.4 and a HR of 3.8 of mortality,^9 31^ while a study of the GLADEL cohort in Latin America reported an OR of 2.8 comparing SDI=0 to SDI>0.^32^ In our study, we observed significant associations between the presence of organ damage assessed by the novel RBODI and mortality, using the same cut-offs as previous investigations, with HRs of 2.1 (RBODI=0 vs RBODI>0) and 2.4 (RBODI≤1 vs RBODI>1). These observations further support the utility of RBODI as a reliable instrument for assessing organ damage in patients with SLE and reinforce the importance of organ damage prevention as a relevant target in the management of SLE.

The development of RBODI opens several avenues for research. First, RBODI can be used as an exposure variable in large-scale investigations of associations with outcomes that are not collected routinely in clinical registers, such as healthcare utilisation, work disability or pregnancy outcomes. Second, RBODI can be considered a measure of morbidity that captures information relevant to SLE populations, complementary to other generic proxies such as the Charlson Comorbidity Index,^33^ number of hospitalisations or drugs dispensed, and thus could be used to adjust for confounding by morbidity status.

Considering that the prevention of organ damage is a major goal in the management of SLE,^1 2^ as well as a component of the emerging concept of disease modification,^34^ one of the most important potential applications of RBODI may be its use as an outcome in studies of interventions in large SLE populations.^35^ In the case of biologics, long-term extension studies of RCTs have been designed to include a subset of patients who completed the double-blind period. Major drawbacks of this study design include selection bias and the lack of a comparator group in most studies.^36^,^38^ Furthermore, the rather low rate of organ damage accrual in SLE populations, in the context of an experimental setting, makes them expensive and time-consuming.^4^ Using a population-based cohort study design, particularly in settings with universal access to healthcare with complete data on the entire population, constitutes an efficient option to address the aforementioned disadvantages and generate more generalisable estimates.

Our study has several strengths. During the translation of SDI items to develop the RBODI, we scrutinised several classification systems and collaborated with practitioners from eight medical specialties, aiming to increase the precision of the definitions and to reflect modern clinical practice in Sweden. We linked several data sources to create the RBODI, allowing us to use diagnosis and procedure data from outpatient and inpatient visits along with medications dispensed to the patients. We relied on classification systems that are used widely, particularly ICD-10 and ATC codes, aiming to develop an index that can be applied in different countries, although its feasibility and generalisability warrant external validation studies in settings outside of Sweden. Given that the National Patient Register provides exact dates for hospitalisation and outpatient visits, RBODI enables identification of events closer to their actual occurrence compared with the natural delay associated with the registration of SDI by a rheumatologist. Also, we described the occurrence of organ damage and its relationship with mortality in a large nationwide sample, which minimises selection bias and increases the external validity of our findings. This sample also allowed us to study associations with mortality over a long follow-up.

During the development of the RBODI, we encountered challenges leading to potential misclassification. First, certain items cannot be adequately captured using codes, including estimated glomerular filtration rate <50% within the range of 30–50 mL/min; proteinuria >3.5 g/24 hours; cognitive impairment that does not correspond to dementia; and muscle atrophy or weakness. Second, despite the existence of codes for certain diagnoses, clinicians may lack incentive to provide them, as evidenced by previous research from Sweden^39^; in our case, this was apparent for the four uncommon diagnoses that we could not detect with codes, along with more frequent conditions such as deforming or erosive arthritis, scarring alopecia and premature gonadal failure. Third, we lacked primary care data, constituting a further reason for misclassification of diagnoses such as diabetes and cognitive impairment, which we mitigated by including information on prescriptions as a part of the definitions. Considering that patients with SLE are in frequent contact with secondary and tertiary care, we expect this misclassification to be minor, as these diagnoses are likely to be recorded in the National Patient Register.

Lastly, RBODI is constructed based on the SDI, which does not fully account for the modern conception of SLE as a disease continuum, where disease progresses from preclinical autoimmunity to an established SLE diagnosis.^3^ Evidence supporting this new paradigm encompasses the presence of autoantibodies—including antiphospholipid antibodies^40 41^—and activation of the interferon pathway^42 43^ years before the SLE diagnosis; the high comorbidity burden prior to and at the time of diagnosis^20 21^; the significant proportion of individuals receiving glucocorticoids before receiving a formal SLE diagnosis; and obstacles in healthcare to obtain a prompt medical appointment with a specialist in the prodromal phase of the disease.^44^ As our index aimed to mirror the SDI, it detects damage occurring from SLE diagnosis. This poses a practical challenge when diagnoses such as stroke, myocardial infarction or thrombotic events occur slightly prior to a definite SLE diagnosis, as they would not be included in the RBODI calculation, leading to misclassification. Although future versions of the SDI are expected to address this limitation by incorporating prediagnostic damage attributable to SLE,^3^ we anticipate that attribution will remain a challenge. Nonetheless, RBODI retains its utility in capturing organ damage developed from diagnosis, and we expect it may provide insights that contribute to the revision of the SDI.^3^

In conclusion, we have developed a novel register-based organ damage index that accurately estimates SDI scores, and have used the index to describe damage accrual over nearly two decades in the largest unselected population of incident SLE to date. Despite the arrival of new drugs designed and approved for SLE and updated clinical guidelines, nearly 40% of newly diagnosed patients with SLE develop organ damage within the first 5 years of diagnosis, with no changes in the risk of organ damage by calendar period. The strong association between organ damage accrual early in the disease course and future mortality highlights the need for treat-to-target strategies that incorporate early and effective interventions to prevent organ damage.

## supplementary material

10.1136/lupus-2024-001403 online supplemental file 1

## Data Availability

Data are available upon reasonable request. Data may be obtained from a third party and are not publicly available.
