# Supplementary material for: Development and evaluation of a Register-Based Organ Damage Index in systemic lupus erythematosus: a nationwide, population-based study from Sweden
Source: Lupus Sci Med. 2025 Feb 26;12(1):e001403. doi: 10.1136/lupus-2024-001403 (PMC11865802; doi:10.1136/lupus-2024-001403)

Development and evaluation of a register-based organ damage index in systemic lupus erythematosus: a nationwide, population-based study from Sweden

Alvaro Gomez[1], Ioannis Parodis[1,2], Muna Saleh[3], Julia F. Simard[4,5], Christopher Sjöwall[3], Elizabeth V. Arkema[5]

[1]Division of Rheumatology, Department of Medicine Solna, Karolinska Institutet and Karolinska University Hospital, Stockholm, Sweden.

[2]Department of Rheumatology, Faculty of Medicine and Health, Örebro University, Örebro, Sweden.

[3]Department of Biomedical and Clinical Sciences, Division of Inflammation and Infection/Rheumatology, Linköping University, Sweden.

[4]Department of Epidemiology and Population Health; Division of Immunology and Rheumatology, Department of Medicine, Stanford University School of Medicine, Stanford, California, USA.

[5]Clinical Epidemiology Division, Department of Medicine Solna, Karolinska Institutet, Stockholm, Sweden.

Supplemental material

Supplemental Table S1. *Item definition for the register-based organ damage index (RBODI)* ***3***

Supplemental Table S2. *Accuracy measurements of RBODI to detect the presence of organ damage related to or independent of glucocorticoid use according to SDI in SLE cases from Östergötland, Sweden 2021 (N=271)* ***22***

Supplemental Table S3. *Cumulative incidence at 5 years after diagnosis for all RBODI organ domains by sex.* ***23***

Supplemental Figure S1. *Flow diagram of participant disposition in the evaluation cohort* ***24***

Supplemental Figure S2. *Study design diagram for the validation of RBODI.* ***25***

Supplemental Figure S3. *Study design diagram for organ damage occurrence in the nationwide cohort of newly diagnosed SLE patients.* ***26***

Supplemental Figure S4. *Study design diagram for the association between organ damage accrual and mortality risk in the nationwide cohort of newly diagnosed SLE patients* ***27***

Supplemental Figure S5. *Cumulative incidence at five years after diagnosis for all organ damage domains in newly diagnosed patients with SLE in Sweden (N=4441).* ***28***

Supplemental Table S1. Item definition for the register-based organ damage index (RBODI)

SDI Item	Clinical diagnoses (ICD-10 codes)	Medical procedures (KVÅ codes)	Surgical procedures (KVÅ and DRG codes)	Prescriptions (ATC codes)
OCULAR				
Any cataract ever	H25, Senile cataract<br>H26, Other cataract<br>H28, Cataract and other disorders of lens in diseases classified elsewhere		CJC, Intracapsular cataract extraction<br>CJD, Extracapsular cataract extraction<br>CJE, Extracapsular cataract extraction with phacoemulsification	
Retinal change OR optic atrophy	H32, Chorioretinal disorders in diseases classified elsewhere<br>H34, Retinal vascular occlusions<br>H35.0 Background retinopathy and retinal vascular changes<br>H35.2, Other proliferative retinopathy<br>H35.3, Degeneration of macula and posterior pole)<br>H35.4, Peripheral retinal degeneration<br>H35.6, Retinal haemorrhage			

	H35.7, Separation of retinal layers<br><br>H35.8, Other specified retinal disorders<br><br>H35.9, Retinal disorder, unspecified<br><br>H36, Retinal disorders in diseases classified elsewhere<br><br>H47.2, Optic atrophy<br><br>H48.0, Optic atrophy in diseases classified elsewhere<br><br>H48.1, Retrobulbar neuritis in diseases classified elsewhere			
NEUROPSYCHIATRIC				
Cognitive impairment (e.g., memory deficit, difficulty with calculation, poor concentration, difficulty in spoken or written language, impaired performance level)<br><br>OR major psychosis	**Cognitive impairment**:<br><br>F00, Dementia in Alzheimer disease<br><br>F01, Vascular dementia<br><br>F02, Dementia in other diseases classified elsewhere<br><br>F03, Unspecified dementia<br><br>F04, Organic amnesic syndrome, not induced by alcohol and other psychoactive substances<br><br>F05.1, Delirium superimposed on dementia<br><br>G30, Alzheimer disease			

	G31.1, Senile degeneration of brain, not elsewhere classified<br><br>G31.8A, Degenerative disorders in Lewy body dementia<br><br>Excluding:<br><br>F70-F79, Mental retardation			
	**Major psychosis**:<br><br>F20, Schizophrenia<br><br>F22, Persistent delusional disorders<br><br>F25, Schizoaffective disorders<br><br>Excluding:<br><br>F70-F79, Mental retardation			**Major psychosis**:<br><br>N05AH02, Clozapine<br><br>[Two or more prescriptions, at least six months apart]
Seizures requiring therapy for 6 months (1)	G40, Epilepsy<br><br>G41, Status epilepticus			N03, Antiepileptics<br><br>[Two or more prescriptions, at least six months apart]<br><br>[Definition requires clinical diagnosis and prescription]
Cerebral vascular accident ever	I60, Subarachnoid haemorrhage			

(score 2 if > 1), or resection not for malignancy (2)	I61, Intracerebral haemorrhage<br><br>I63, Cerebral infarction<br><br>I64, Stroke, not specified as haemorrhage or infarction<br><br>G45, Transient cerebral ischaemic attacks and related syndromes			
Cranial or peripheral neuropathy (excluding optic)	E11.4, Type 2 diabetes mellitus – with neurological complications<br><br>G50, Disorders of trigeminal nerve<br><br>G51, Facial nerve disorders<br><br>G52, Disorders of other cranial nerves<br><br>G53, Cranial nerve disorders in diseases classified elsewhere<br><br>G57.2, Lesion of femoral nerve<br><br>G57.3, Lesion of lateral popliteal nerve<br>G57.4, Lesion of medial popliteal nerve<br><br>G58.7, Mononeuritis multiplex<br><br>G58.8, Other specified mononeuropathies<br><br>G58.9, Mononeuropathy, unspecified<br><br>G59, Mononeuropathy in diseases classified elsewhere			

	G61, Inflammatory polyneuropathy<br><br>G62, Other polyneuropathies<br><br>G63, Polyneuropathy in diseases classified elsewhere<br><br>G64, Other disorders of peripheral nervous system			
Transverse myelitis	G37.3, Acute transverse myelitis in demyelinating disease of central nervous system			
RENAL				
Estimated or measured GFR <50 %	N18.4, Chronic kidney disease, stage 4			
Proteinuria 24 h >3.5 g<br>OR<br>End-stage renal disease (regardless of dialysis or transplantation)	**ESRD**:<br><br>N18.5, Chronic kidney disease, stage 5<br><br>**Renal transplant**:<br><br>Z94.0, Kidney transplant status<br><br>**Renal dialysis**:<br><br>Z99.2, Dependence on renal dialysis	**Renal dialysis**:<br><br>DR016, Haemodialysis, chronic<br><br>DR024, Peritoneal dialysis, chronic<br><br>DR061, Start of haemodialysis	**Renal transplant**:<br><br>KAS00, Autologous kidney transplantation<br><br>KAS10, Open transplantation of kidney from deceased donor<br><br>KAS20, Open transplantation of kidney from living donor	
PULMONARY				

Pulmonary hypertension (right ventricular prominence, or loud P2) (4)	I27.0, Primary pulmonary hypertension<br><br>I27.2, Other secondary pulmonary hypertension<br><br>I27.9, Pulmonary heart disease, unspecified			
Pulmonary fibrosis (physical and X-ray)	J70.1, Chronic and other pulmonary manifestations due to radiation<br><br>J70.3, Chronic drug-induced interstitial lung disorders<br><br>J84.1, Other interstitial pulmonary diseases with fibrosis			
Shrinking lung (X-ray)				
Pleural fibrosis (X-ray)	J92, Pleural plaque<br><br>J94.1, Fibrothorax			
Pulmonary infarction (X-ray) OR resection not for malignancy	I26, Pulmonary embolism		GDB, Lung resection excluding C34, Malignant neoplasm of bronchus and lung	
CARDIOVASCULAR				
Angina OR coronary artery bypass	**Angina**<br><br>I20, Angina pectoris<br><br>I24, Other acute ischaemic heart diseases I25, Chronic ischaemic heart disease			

	**CABG**<br><br>Z95.1, Presence of aortocoronary bypass graft<br><br>Z95.5, Presence of coronary angioplasty implant and graft		**CABG**<br><br>FNA, Anastomosis between internal thoracic artery and coronary arteries<br><br>FNB, Anastomosis between gastroepiploic artery and coronary arteries<br><br>FNC, Aortocoronary vein bypass<br><br>FND, Aortocoronary bypass with vascular prosthesis<br><br>FNE, Coronary bypass with free artery grafts<br><br>FNG, Dilatation and recanalisation of coronary artery	
Myocardial infarction ever (score 2 if > 1) (5)	I21, Acute myocardial infarction<br><br>I22, Subsequent myocardial infarction			
Cardiomyopathy (ventricular dysfunction)	I25.5, Ischaemic cardiomyopathy<br><br>I42, Cardiomyopathy			
Valvular disease (diastolic murmur,	I05, Rheumatic mitral valve diseases<br><br>I06, Rheumatic aortic valve diseases		FG, Tricuspid valve surgery	

or a systolic murmur > 3/6)	I07, Rheumatic tricuspid valve diseases<br><br>I08, Multiple valve diseases<br><br>I34, Nonrheumatic mitral valve disorders<br><br>I35, Nonrheumatic aortic valve disorders<br><br>I36, Nonrheumatic tricuspid valve disorders<br><br>I37, Pulmonary valve disorders<br><br>Z95.2, Presence of prosthetic heart valve<br><br>Z95.3, Presence of xenogeneic heart valve<br><br>Z95.4, Presence of other heart-valve replacement		FK, Mitral valve surgery<br><br>FM, Aortic valve surgery<br><br>FJE, Surgery for isolated pulmonary stenosis<br><br>FJF, Implantation of pulmonary valve prosthesis	
Pericarditis x 6 months or pericardiectomy	I09.2, Chronic rheumatic pericarditis<br><br>I31.0, Chronic adhesive pericarditis<br><br>I31.1, Chronic constrictive pericarditis		FEB, Pericardiotomy<br><br>FEF, Resection and extirpation of the pericardium	
PERIPHERAL VASCULAR				
Claudication x 6 months	I73.9B, Intermittent claudication			B01AC23, cilostazol<br><br>C04AD03, pentoxifylline<br><br>[Two or more prescriptions, at least six months apart]

Minor tissue loss (pulp space)	I73.9C Digital ischaemia			
Significant tissue loss ever (e.g., loss of digit or limb, resection) (score 2 if > 1)			[KVÅ]<br><br>NHQ, Amputation and related operations on ankles and feet<br><br>[DRG]<br><br>E20, 113, 114, Amputations due to circulatory diseases, other than upper limb or toe<br><br>E21, Upper limb or toe amputations due to circulatory diseases<br><br>H09, Amputation for muscle, bone or connective tissue disorders<br><br>L01, Lower limb amputation for endocrine, nutritional & metabolic diseases	
Venous thrombosis with swelling,	I80, Phlebitis and thrombophlebitis<br><br>I81, Portal vein thrombosis			

ulceration, OR venous stasis (6)	I82, Other venous embolism and thrombosis			
GASTROINTESTINAL				
Infarction or resection of bowel (below duodenum), spleen, liver or gall bladder ever (score 2 if >1)	**Bowel:**<br><br>K55.0, Acute vascular disorders of intestine<br><br>**Spleen:**<br><br>D73.5, Infarction of spleen<br><br>**Liver:**<br><br>K76.3, Infarction of liver		**Bowel:**<br><br>[KVÅ]<br><br>JFB, Resection of small bowel and colon<br><br>JFH, Colectomy<br><br>[DRG]<br><br>F01, 146, 147, Rectal resection and extirpation<br><br>F05, 148, 149, Major small and large bowel surgery<br><br>F09, 152, 153, Minor small and large bowel surgery<br><br>F30, 166, 167, Appendectomy<br><br>**Spleen:**<br><br>[KVÅ]<br><br>JMA, Spleen resection and splenectomy	

			[DRG]<br><br>R01N, 392, 393O, Spleen extirpation<br><br><br>**Liver:**<br><br>[KVÅ]<br><br>JJB, Liver resection<br><br>[DRG]<br><br>G01N, 480, Liver transplant<br><br><br>**Gallbladder:**<br><br>[KVÅ]<br><br>JKA20, Cholecystectomy<br><br>JKA21, Laparoscopic cholecystectomy<br><br>[DRG]<br><br>G10, 196, 196, Cholecystectomy with coledochal exploration<br><br>G11, 197, 198, Open cholecystectomy	

			without coledochal exploration<br><br>G12, 493, 494, Laparo/endoscopic biliary surgery<br><br>G30, 4940, Cholecystectomy and biliary tract surgery	
Mesenteric insufficiency	K55.1, Chronic vascular disorders of intestine			
Chronic peritonitis	K65.8, Other peritonitis<br><br>N73.4, Female chronic pelvic peritonitis			
Stricture OR upper gastrointestinal tract surgery ever	K22.2, Oesophageal obstruction		[KVÅ]<br><br>JC, Oesophageal surgery<br><br>JD, Stomach and duodenum surgery<br><br>[DRG]<br><br>L08, 288, Gastrointestinal surgery for obesity<br><br>F11, 154–156, Major surgery of the stomach, oesophagus and duodenum<br><br>F12, Other operations of the stomach,	

			oesophagus and duodenum<br><br>F13O, Surgery of the stomach, oesophagus and duodenum<br><br>F35, 17O, 171 Other operations for gastrointestinal diseases	
Pancreatic insufficiency requiring enzyme replacement or with pseudocyst	K86.0, Alcohol-induced chronic pancreatitis<br><br>K86.1, Other chronic pancreatitis<br><br>K86.3, Pseudocyst of pancreas<br><br>K86.8, Other specified diseases of pancreas<br><br>K86.9, Disease of pancreas, unspecified<br><br>E16.9, Disorder of pancreatic internal secretion, unspecified			A09AA02, multienzymes (lipase, protease, etc)<br><br>[Definition requires clinical diagnosis or prescription + K86.8, K86.9, K90.3, E16.9]
MUSCULOSKELETAL				
Atrophy or weakness	M62.5, Muscle wasting and atrophy, not elsewhere classified			
Deforming or erosive arthritis (including reducible deformities, excluding	M12.0, Chronic postrheumatic arthropathy [Jaccoud]			

avascular necrosis)	M05.8L Erosive rheumatoid arthritis, RF-positive and ACPA-positive,<br><br>M05.8M Erosive rheumatoid arthritis, RF-positive and ACPA-negative<br><br>M05.8N Erosive rheumatoid arthritis, RF-positive and ACPA-unspecified<br><br><br>M06.8L Erosive rheumatoid arthritis, RF-negative and ACPA-positive,<br><br>M06.8M Erosive rheumatoid arthritis, RF-negative and ACPA-positive,<br><br>M06.8N Erosive rheumatoid arthritis, RF-negative and ACPA-positive			
Osteoporosis with fracture or vertebral collapse (excluding avascular necrosis) (7)	**Osteoporosis:**<br><br>M80, Osteoporosis with pathological fracture<br><br>M81, Osteoporosis without pathological fracture<br><br>M82, Osteoporosis in diseases classified elsewhere<br><br><br>**Non-vertebral fracture:**<br><br>S12, Fracture of neck<br><br>S22, Fracture of rib(s), sternum and thoracic spine			G03XC01, Tibolone<br><br>H05AA02, Teriparatide<br><br>M05BA, Bisphosphonates<br><br>M05BB, Bisphosphonates combinations<br><br>M05BX03, Strontium ranelate<br><br>M05BX04, Denosumab<br><br>[Two or more prescriptions, at least six months apart]

	S32, Fracture of lumbar spine and pelvis<br><br>S42, Fracture of shoulder and upper arm<br><br>S52, Fracture of forearm<br><br>S62, Fracture at wrist and hand level<br><br>S72, Fracture of femur<br><br>S82, Fracture of lower leg, including ankle<br><br>S92, Fracture of foot, except ankle<br><br>T02, Fractures involving multiple body regions<br><br>T08, Fracture of spine, level unspecified<br><br>T10, Fracture of upper limb, level unspecified<br><br>T12, Fracture of lower limb, level unspecified<br><br>T14.2, Fracture of unspecified body region<br><br><br>**Vertebral collapse:**<br><br>M48.5, Collapsed vertebra, not elsewhere classified<br><br>S12.0, Fracture of first cervical vertebra<br><br>S12.1, Fracture of second cervical vertebra<br><br>S12.2, Fracture of other specified cervical vertebra			

	S22.0, Fracture of thoracic vertebra<br><br>S22.1, Multiple fractures of thoracic spine<br><br>S32.0, Fracture of lumbar vertebra<br><br>[Definition requires a code for osteoporosis and at least one code for non-vertebral fracture or vertebral collapse].			
Avascular necrosis (score 2 if > 1)	M87.0, Idiopathic aseptic necrosis of bone<br><br>M87.3, Other secondary osteonecrosis<br><br>M87.8, Other osteonecrosis<br><br>M87.9, Osteonecrosis, unspecified			
Osteomyelitis	M86, Osteomyelitis			
Ruptured tendons	M66.2, Spontaneous rupture of extensor tendons<br><br>M66.3, Spontaneous rupture of flexor tendons<br><br>M66.4, Spontaneous rupture of other tendons<br><br>M66.5, Spontaneous rupture of unspecified tendon			
SKIN				

Alopecia	L66, Cicatricial alopecia [scarring hair loss]			
Extensive scarring or paniculum other than scalp and pulp space	L90.5, Scar conditions and fibrosis of skin			
Skin ulceration (excluding thrombosis) for more than 6 months	E10.6D, Type 1 diabetes mellitus with foot ulcer<br>E11.6D, Type 2 diabetes mellitus with foot ulcer<br>I70.2C, Atherosclerosis of native arteries of the extremities with ulcer<br>I83.0, Varicose veins of lower extremities with ulcer<br>I83.2, Varicose veins of lower extremities with both ulcer and inflammation<br>L89, Pressure ulcer<br>L97, Ulcer of lower limb, not elsewhere classified<br>[Two or more codes, at least six months apart]<br>L98.4, Chronic ulcer of skin, not elsewhere classified			

PREMATURE GONADAL FAILURE (8)	E28.3, Primary ovarian failure<br><br>E89.4, Postprocedural ovarian failure			Prescription to women younger than 40<br><br>G03CA03, oestradiol<br><br>G03CA57, conjugated oestrogens<br><br>G03CX01, tibolone<br><br>G03FA01, norethisterone and oestrogen<br><br>G03FA12 medroxyprogesterone and oestrogen<br><br>G03FA15, dienogest and oestrogen<br><br>G03FA17, drospirenone and oestrogen<br><br>G03FB05, norethisterone and oestrogen<br><br>G03FB06 medroxyprogestgerone<br><br>and oestrogen<br><br>G03FB09 levonorgestrel and oestrogen

				[Two or more prescriptions, at least six months apart]
DIABETES	E10, Type 1 diabetes mellitus<br><br>E11, Type 2 diabetes mellitus<br><br>E12, Malnutrition-related diabetes mellitus<br><br>E13, Other specified diabetes mellitus<br><br>E14, Unspecified diabetes mellitus			A10, Drugs used in diabetes (9)<br><br>[Two or more prescriptions, at least six months apart]
MALIGNANCY (exclude dysplasia) (9)	C00-C97, Malignant neoplasms (excluding C44, Other malignant neoplasms of skin)<br><br>D00-D09, In situ neoplasms			

Supplemental Table S2. Accuracy measurements of RBODI to detect the presence of organ damage related to or independent of glucocorticoid use according to SDI in SLE cases from Östergötland, Sweden 2021 (N=271).

Item	RBODI, n (%)	SDI, n (%)	Se (95% CI)	Sp (95% CI)	PPV (95% CI)	NPV (95% CI)
GC-related	129 (47.6)	142 (52.4)	0.80 (0.73, 0.86)	0.88 (0.82, 0.93)	0.88 (0.82, 0.93)	0.80 (0.73, 0.86)
GC-definite	80 (29.5)	83 (30.6)	0.76 (0.65, 0.85)	0.91 (0.86, 0.95)	0.79 (0.68, 0.87)	0.90 (0.84, 0.93)
GC-possible	85 (31.4)	109 (40.2)	0.69 (0.59, 0.77)	0.94 (0.89, 0.97)	0.88 (0.79, 0.94)	0.82 (0.75, 0.94)
GC-independent	69 (25.5)	98 (36.2)	0.61 (0.51, 0.71)	0.95 (0.90, 0.98)	0.87 (0.77, 0.94)	0.81 (0.75, 0.86)

GC-definite domains: ocular, musculoskeletal.

GC-possible domains: cardiovascular, peripheral vascular, neuropsychiatric, diabetes.

GC-independent domains: renal, pulmonary, gastrointestinal, skin, gonadal, malignancy.

GC, glucocorticoid; NPV, negative predictive value; PPV, positive predictive value; RBODI, register-based organ damage index; SDI, SLICC/ACR damage index; Se, sensitivity; SLE, systemic lupus erythematosus; Sp, specificity.

Supplemental Table S3. Cumulative incidence at 5 years after diagnosis for all RBODI organ domains by sex.

	**Overall (N=4441)**	**Female (N=3661)**	**Male (N=780)**
Ocular	0.140 (0.129–0.152)	0.128 (0.116-0.139)	0.202 (0.170-0.233)
Neuropsychiatric	0.087 (0.078–0.096)	0.074 (0.065-0.083)	0.116 (0.091-0.141)
Renal	0.025 (0.021–0.030)	0.020 (0.016-0.025)	0.050 (0.033-0.066)
Pulmonary	0.047 (0.040-0.053)	0.045 (0.037-0.052)	0.056 (0.038-0.073)
Cardiovascular	0.079 (0.070–0.087)	0.064 (0.056-0.073)	0.148 (0.121-0.175)
Peripheral vascular	0.046 (0.039–0.052)	0.039 (0.033-0.046)	0.075 (0.055-0.095)
Gastrointestinal	0.047 (0.040–0.054)	0.045 (0.038-0.053)	0.054 (0.036-0.072)
Musculoskeletal	0.043 (0.036–0.049)	0.043 (0.036-0.050)	0.041 (0.025-0.057)
Mucocutaneous	0.006 (0.004–0.009)	0.008 (0.005-0.011)	0.010 (0.003-0.018)
Premature gonadal failure	0.002 (0.001–0.003)	0.002 (0.001-0.004)	N/A
Diabetes	0.047 (0.041–0.054)	0.042 (0.035-0.049)	0.073 (0.054-0.093)
Malignancy	0.068 (0.060–0.076)	0.063 (0.055-0.072)	0.090 (0.067-0.112)

Supplemental Figure S1. Flow diagram of participant disposition in the evaluation cohort

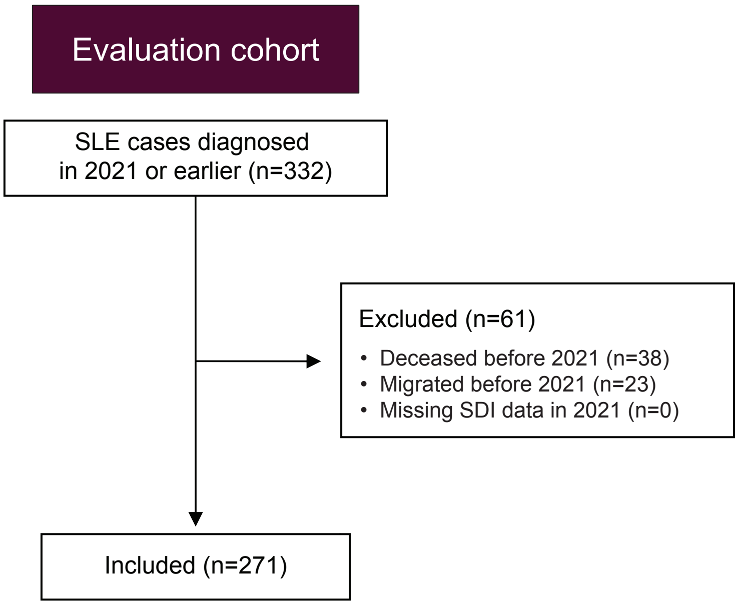

Supplemental Figure S2. Study design diagram for the validation of RBODI.

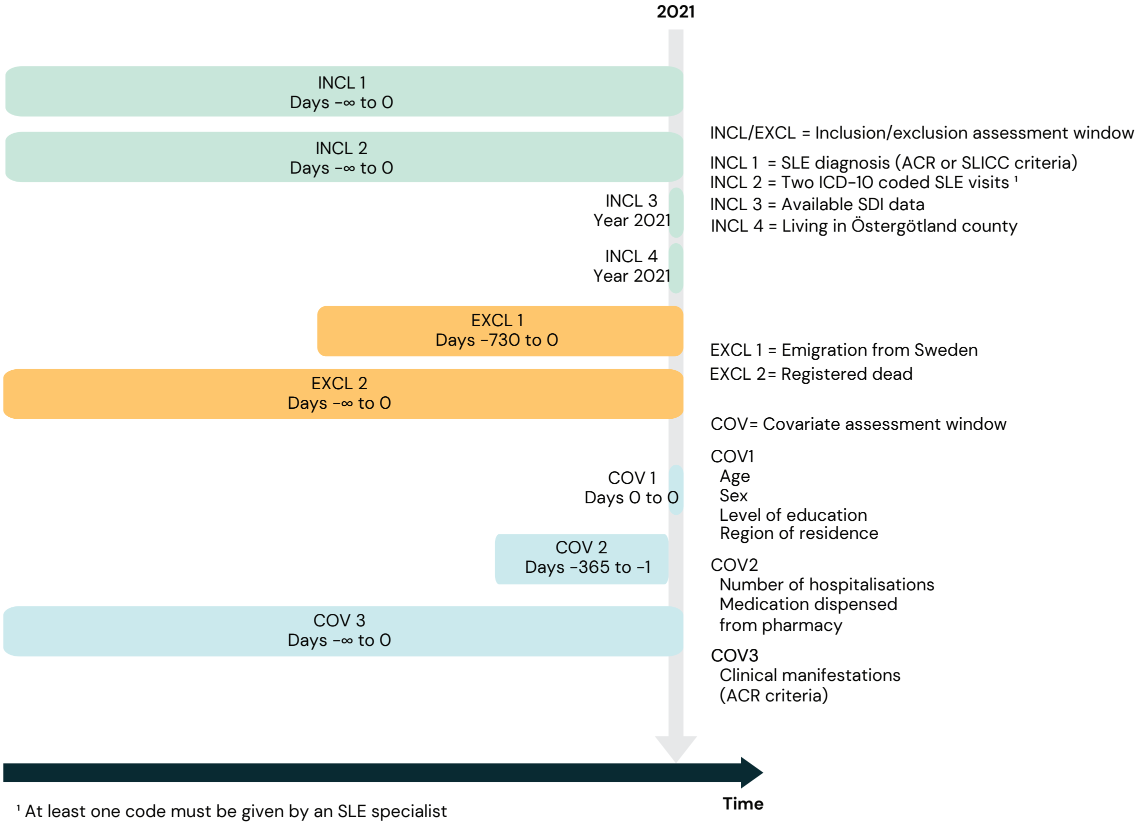

[1] At least one code must be given by an SLE specialist

Supplemental Figure S3. Study design diagram for organ damage occurrence in the nationwide cohort of newly diagnosed SLE patients.

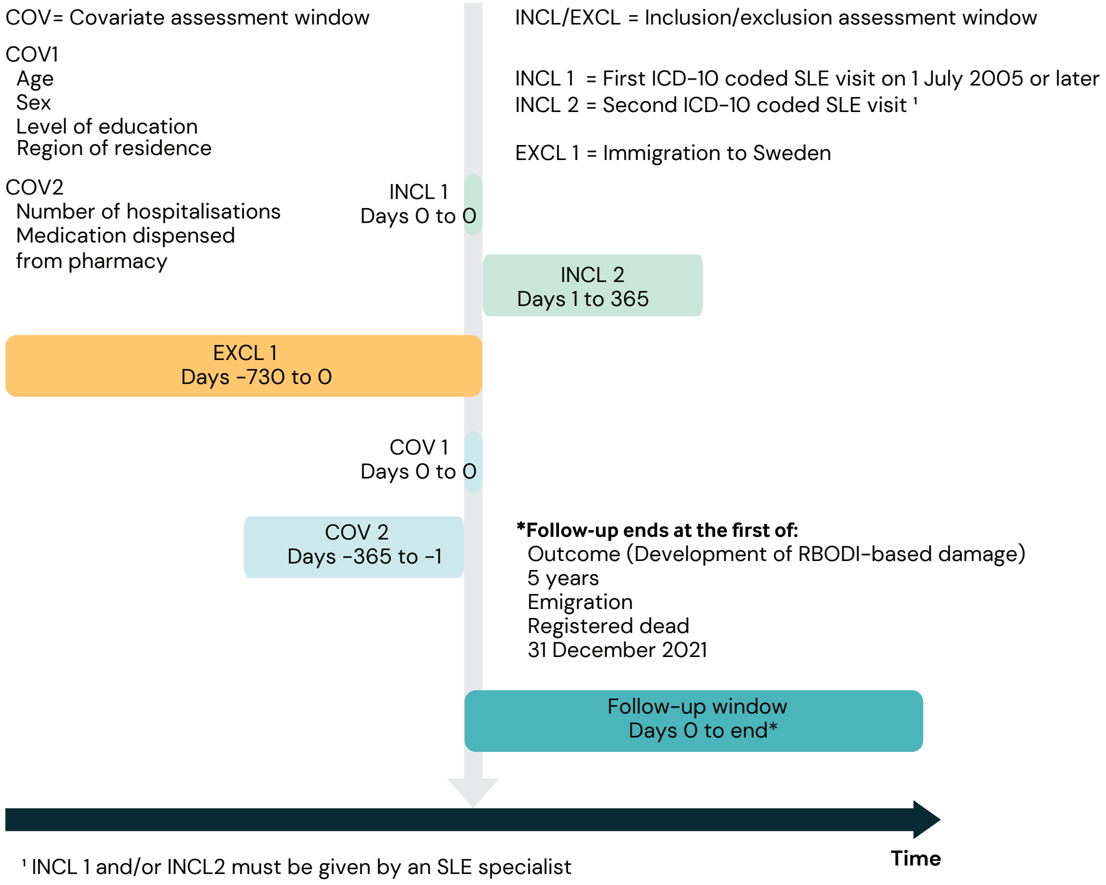

Supplemental Figure S4. Study design diagram for the association between organ damage accrual and mortality risk in the nationwide cohort of newly diagnosed SLE patients.

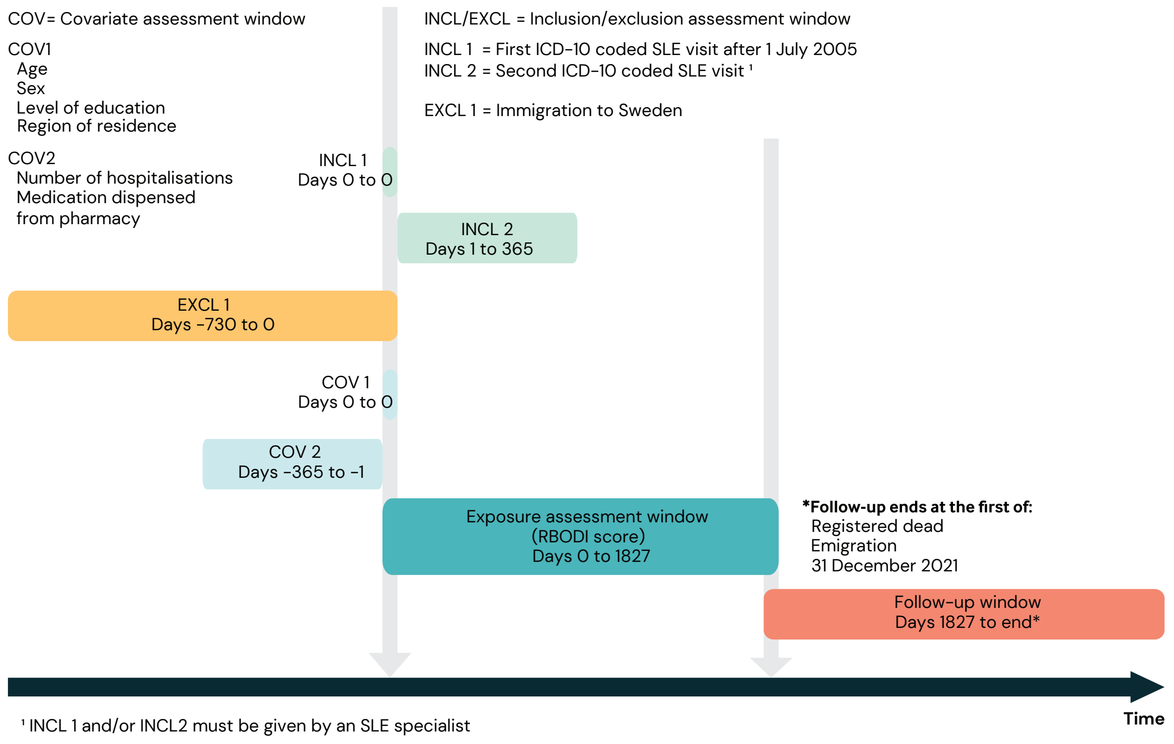

Supplemental Figure S5. Cumulative incidence at five years after diagnosis for all organ damage domains in newly diagnosed patients with SLE in Sweden (N=4441).

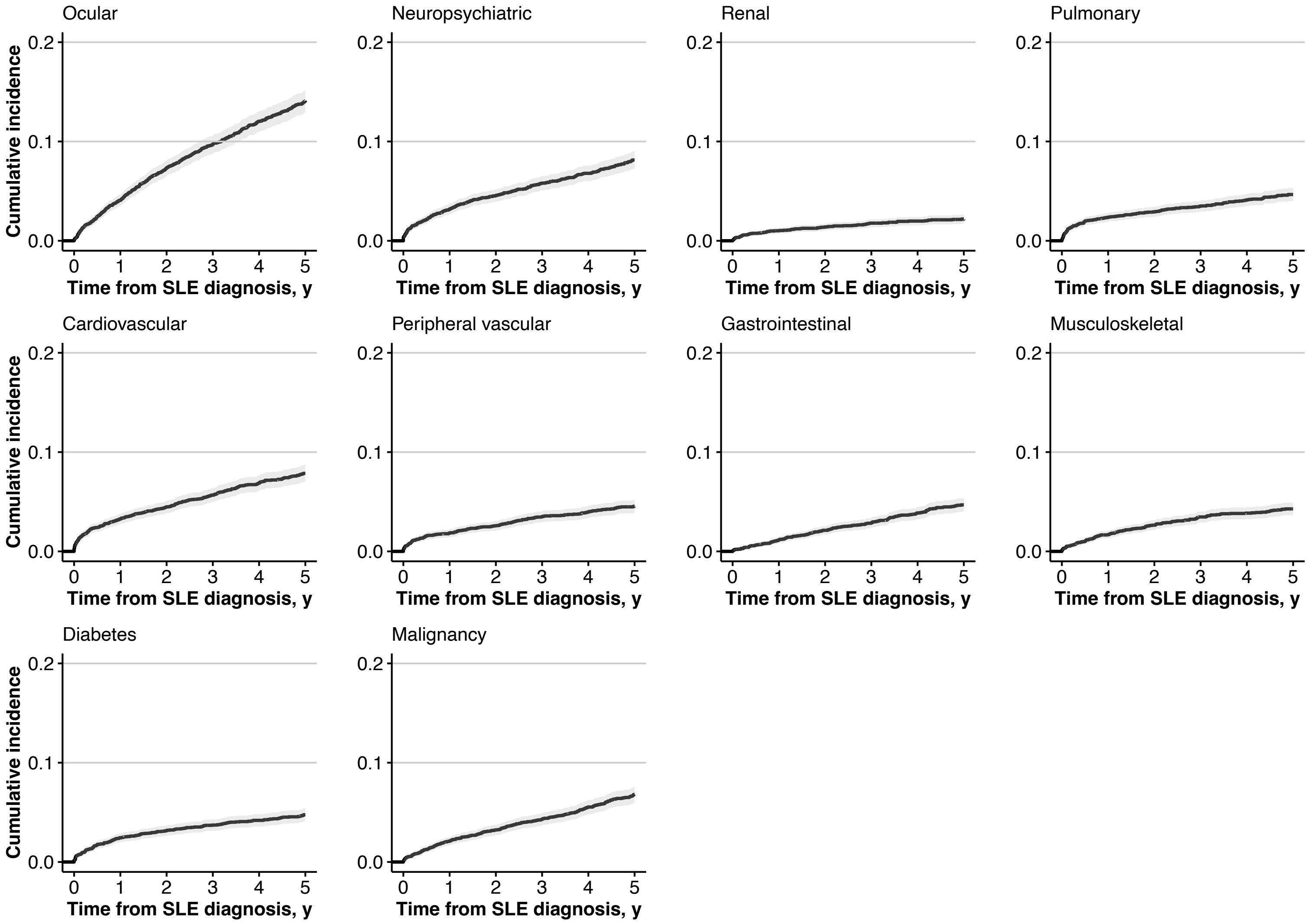

Supplement: online supplemental file 1 [file lupus-12-1-s001.pdf]
